# An explainable Grey Wolf optimized extreme learning machine framework for modulation classification in cloud environment

**DOI:** 10.1038/s41598-026-49723-5

**Published:** 2026-04-27

**Authors:** Padma Charan Sahu, Bibhu Prasad, Ratnakar Dash, Debendra Muduli, Suddhendu DasMahapatra, Siba Mishra, Sourav Parija

**Affiliations:** 1 Department of Electronics and Communication Engineering, GIET University, Gunupur, Odisha India; 2 Department of Computer Science and Engineering, National Institute of Technology Rourkela, Rourkela, Odisha India; 3 Department of Computer Science and Engineering, C.V. Raman Global University, Bhubaneswar, Odisha India; 4 Department of Electronics & Communication Engineering, Manipal University Jaipur, Jaipur, Rajasthan 303007 India

**Keywords:** Automatic modulation classification, Deep feature fusion, Extreme learning machine, Grey Wolf optimization, Explainable artificial intelligence, Engineering, Mathematics and computing

## Abstract

Automatic modulation classification (AMC) is a key component in modern wireless communication systems, supporting efficient spectrum utilization and reliable data transmission. This paper presents a novel AMC model named as XGEM-Net, an explainable grey wolf optimized extreme learning machine model designed for cloud-centric environment. In the proposed model, the features have been extracted from pre-trained models like InceptionV3, ResNet-50, and MobileNetV2 and concatenated to form a unified feature vector. The combined features are input into an ELM optimized via Grey Wolf Optimization (GWO), forming the GWO-ELM model to classify various modulation types. Furthermore, the framework integrates explainable artificial intelligence using local interpretable model agnostic explanations (LIME) to deliver clear, instance level interpretability of classifier decisions. The proposed model has been evaluated in both standalone and cloud-based environments, including configurations with vCPU-4 (16 GB RAM), vCPU-8 (32 GB RAM), and vCPU-16 (64 GB RAM). Experimental results indicate that the model performs significantly better in the cloud setting, with the vCPU-16 64 GB configuration achieving 95.16% accuracy, 90.78% sensitivity, and 89.83% specificity. These findings demonstrate that the proposed approach consistently outperforms existing state-of-the-art methods in terms of classification accuracy.

## Introduction

Automatic Modulation Classification (AMC) is an important part of signal processing^1,2^. This technique is predominantly utilised in military and corporate contexts, particularly in Software Defined Radio (SDR), to swiftly discern modulation types^4,5^. Adaptive and cognitive radio systems are becoming more common, and transmitters need to quickly adapt to changes in the spectrum. This makes an accurate AMC engine essential for real-time spectrum awareness. Also, the rise of different wireless standards in crowded frequency bands means that modulation schemes need to be automatically identified without regard to protocol in order to reduce interference and allow dynamic spectrum access. In modern electronic warfare, dependable AMC is the foundation for threat detection and signal intelligence. In civilian settings, it helps with effective spectrum monitoring to meet regulations and improve network performance. These factors collectively drive continued research into resilient, data-driven AMC methodologies that can function under low signal-to-noise ratios and significant channel impairments. There are generally two primary categories of AMC methodologies. Some methods are based on likelihood, while others are based on features^8^. The likelihood-based method determines the modulation type by contrasting its function with a predefined set of known modulation types^6^. This method is important in multi-channel settings because it uses the modulation classification technique^9^. Adding new things like signal frequency, channel characteristics, and coding rate could make the process a lot harder^10^. In some situations, this method might not work because it makes things more complicated. The feature-based approach extracts traits from the incoming signal. A pattern recognition system uses the features that were collected to figure out how the signal is modulated^12,13^. In traditional pattern recognition algorithms, it is necessary to manually extract particular signal features, including asynchronous delay sampling characteristics, high-order statistics, and time-frequency statistics^14^. After that, the parameters are combined and used to sort things into groups using support vector machines and decision trees^20^. These classifiers are simple to use and quick to compute, but they don’t always work well when the data isn’t linear. There has been a lot of progress in AI lately, and individual chips have become much more powerful. This has made it possible to classify modulation more accurately^21,22^. To reach this goal, it has been very important to use deep learning methods. Deep architecture’s complex structure, which has many layers, can extract extra signal attributes through feature integration, so there is no need for extensive manual data feature selection^24,25^. MobileNet V2, CNN^11^, Inception V3^9^, and Xception are some of the best models that have been used to classify modulation. The Extreme Learning Machine (ELM) sorts the features that it gets from the data. Despite notable progress, existing AMC approaches still face several practical limitations. First, end-to-end deep models often provide strong accuracy but require substantial computational resources. Second, single-backbone or handcrafted feature extraction may fail to capture complementary modulation patterns under noisy conditions. Third, many existing methods operate as black boxes and provide limited interpretability for engineering validation. Fourth, cloud deployment is often treated only as an execution environment rather than being analyzed in terms of scalability and cost-performance tradeoffs. Accordingly, the problem addressed in this work is the design of an AMC framework that is accurate, computationally efficient, interpretable, and deployment-aware. The proposed XGEM-Net alleviates prior limitations by combining fused deep representations, GWO-assisted ELM optimization, and LIME-based explanation. The models make feature vectors that the Extreme Learning Machine (ELM) uses to sort things. We have contributed the following points :

1. proposed XGEM-Net, an Explainable Grey Wolf Optimized ELM model for modulation classification in cloud environments.
2. proposed a unified deep-feature fusion technique that concatenates embeddings from Inception V3, ResNet-50, and MobileNet V2, thereby amplifying modulation-specific discriminative power.
3. proposed a Grey Wolf Optimization–tuned Extreme Learning Machine (GWO-ELM) that achieves rapid, resource-frugal training while outperforming deeper networks in classification accuracy.
4. proposed a LIME driven explainability module that yields instance level visual insights, making each AMC decision transparent and engineer-verifiable.
5. proposed a comprehensive edge-to-cloud evaluation showing near-linear scalability and a peak 95.16% accuracy on a 16-vCPU cloud setup, surpassing state-of-the-art AMC baselines.
6. provide a deployment study that quantifies accuracy, training time, inference throughput, and cost across edge and EC2 configurations, clarifying when cloud execution is beneficial.

The following is how this manuscript is put together. Section 2 surveys the related work, laying the theoretical and methodological groundwork. Section 3 then introduces the proposed XGEM-Net Model in detail. Next, Section 4 describes the research materials and methods, including the complete experimental setup. Section 5 reports and discusses the empirical findings, while Section 6 complements these results with an explainable-AI analysis. Finally, Section 7 summarises the principal insights and draws the main conclusions.

## Related Work

### Classical AMC methods

Modulation classification is of considerable importance in wireless communication. This task generally comprises three primary steps: feature extraction, classifier creation, and training of the learning model. Image classification in classical machine learning has extensive applications^26^. Conventional techniques for image classification depend on the extraction of individual or clusters of features utilizing Support Vector Machines. Ali et al.^31^ proposed an innovative approach for precise modulation classification that incorporates ANN-PCA for sample generation and leverages normalization for feature extraction, yielding a normalized support vector.

### Deep learning for AMC

Contemporary Artificial Neural Network (ANN) classifiers provide extensive feature extraction capabilities. Deep learning employs multilayer network models trained on extensive datasets, facilitating layer-by-layer feature extraction to capture advanced image characteristics. In addition to capturing the essential elements of an image, deep learning network models can extract profound features via numerous hidden layers. These deep learning capabilities provide superior accuracy relative to conventional machine learning techniques and are also more efficacious in image classification. Zeng et al.^25^ presented a convolutional neural network model for the automatic recognition of various modulations. Y. Liu et al.^28^ developed a deep learning model explicitly intended for categorization tasks. The deep learning model is crucial in ascertaining the manner in which features are acquired and assimilated during the processes of picture identification and classification^29^. Recent studies across multiple domains, especially in picture classification, indicate a progressive transition from conventional methods to deep learning models^30^. Chen et al.^32^ devised a deep multi-scale convolutional neural network to augment the resilience of recognition capabilities in varying SNR conditions. Sun et al.^33^ presented the Inception-ResNet network, tailoring modulation categorization for software-defined radio through alterations in kernel sizes. Zhang et al.^28^ addressed the problem of overfitting in modulation classification with a digital modulation classifier employing a student-mentor network architecture. Zhou et al.^34^ converted raw signal data into tensors for compatibility with deep learning frameworks. Yin et al.^35^ advocated for the offline pre-training of CNNs with a sufficient sample size prior to deployment, rendering their approach independent of SNR estimation. Sathyanarayanan et al.^36^conducted a comparative analysis of basic CNN and residual CNN to assess their performance accuracy. The study of driver emotion recognition system by Zaman et al^52,53^. showed the usage of neural networks and ensemble classification.

### Feature fusion and transfer learning

Recent studies have demonstrated the effectiveness of deep learning–based feature fusion and ensemble classification techniques for intelligent signal processing and human–robot interaction systems^49,50^. Beyond AMC, recent work in consumer and edge intelligence^43^ shows that careful feature extraction and model compression can significantly improve deployability. Knowledge distillation has been used to build lightweight multimedia anomaly and integrity detectors for consumer IoT while retaining accuracy. Privacy-preserving feature learning has also been explored in decentralized recommendation systems, demonstrating that useful representations can be learned without centralizing user data^43,44^. Hybrid feature extraction pipelines combining optimized RNNs with tracking have been reported for robust motion detection under occlusion. These directions motivate our emphasis on compact backbones, fused embeddings, and deployment-aware evaluation.

### Optimization and explainability

Recent work on lightweight multimedia anomaly and integrity detection for consumer IoT has shown that knowledge distillation can significantly improve deployability while preserving predictive performance^51^. This direction is relevant to the present study because it highlights the importance of compact yet expressive feature extraction in resource-aware intelligent systems.

### Research gap

Existing AMC studies largely fall into two camps: end-to-end deep networks that deliver high accuracy but at the cost of heavy training overhead, and lightweight machine-learning pipelines whose hand-crafted or single-CNN features struggle with complex, noisy spectra. A comparative overview of the advantages and limitations of representative AMC approaches is summarized in Table 1, while Table 2 contrasts different feature representation strategies used in AMC systems. Building on these insights, our work positions itself at the intersection of efficiency and performance: we aggregate complementary embeddings from three compact yet diverse CNN backbones to form a rich, unified representation, then exploit a Grey Wolf-optimized Extreme Learning Machine to achieve rapid, resource-frugal classification. To bridge the transparency gap identified in prior literature, we weave LIME into the workflow, enabling instance-level visual rationales for every prediction—a capability rarely offered in AMC research. Finally, by benchmarking on both edge hardware and scaled cloud instances, we provide the first systematic evidence that a hybrid deep-feature with GWO-ELM approach can attain state-of-the-art accuracy while remaining practical across a wide spectrum of deployment budgets.

**Table 1:** Advantages and limitations of representative prior AMC approaches.

Method category	Advantages	Limitations
Likelihood-based AMC	Strong theoretical foundation	High computational cost and sensitivity to unknown parameters
Handcrafted feature-based AMC	Lower complexity	Limited robustness in noisy conditions
Single-backbone deep CNNs	Strong representation learning	Limited feature diversity
End-to-end hybrid deep models	High predictive accuracy	Heavy training overhead and low interpretability
ELM-based classifiers	Fast training	Sensitive to hidden-layer configuration
Optimization-assisted ELM	Better parameter selection	Extra search overhead and often limited explainability

**Table 2:** Comparison of representation strategies in AMC systems.

Strategy	Representation source	Strength	Limitation
Handcrafted statistics	Moments, cumulants, cyclic features	Low computational cost	Limited adaptability
Single CNN features	One deep backbone	Strong learned representation	Limited diversity
Classical feature selection	Wrapper/filter selection on engineered features	Reduced dimensionality	Depends on initial feature pool
Proposed deep feature fusion	InceptionV3 + ResNet-50 + MobileNetV2	Complementary discriminative information	Slightly higher extraction overhead

## Proposed XGEM-net model

### Overall architecture of the proposed model

**Fig. 1:**
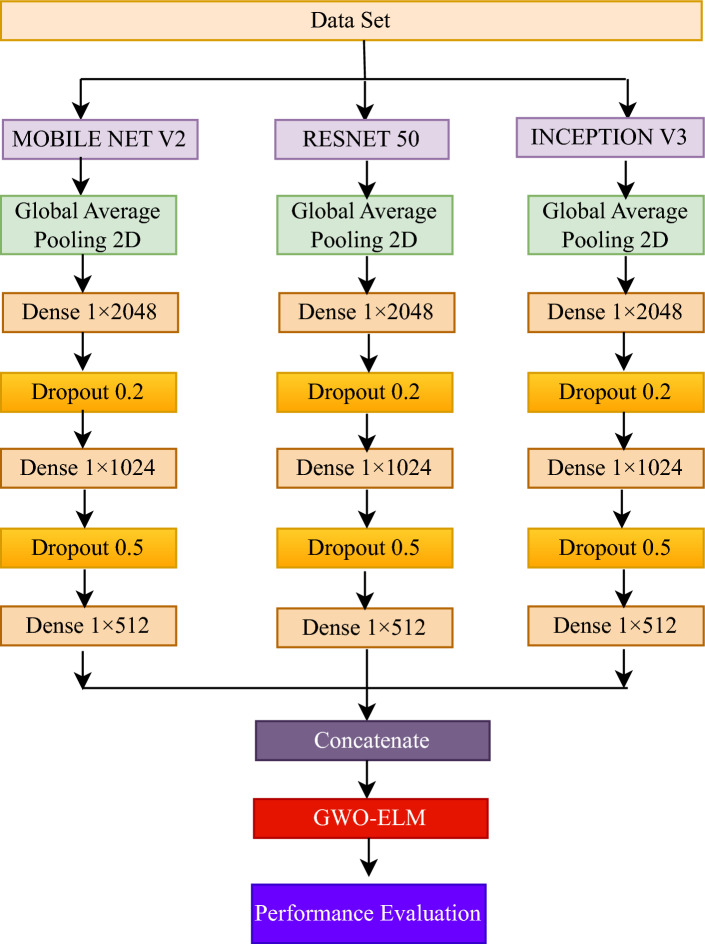
Proposed Network Model.

In this study we introduce **XGEM-Net**—an *Explainable Grey-Wolf-Optimised Extreme Learning Machine* (GWO–ELM) model tailored for automatic modulation classification in cloud environments. The first step is to turn the received complex baseband signal into a two-dimensional polar image. This step makes unique geometric patterns stand out, which makes predictions more reliable. Three complementary, ImageNet-pre-trained backbones—MobileNetV2, ResNet-50, and Inception-V3—get the resulting images at the same time. At the end of each branch is Global Average Pooling, followed by three fully connected layers (2048$\rightarrow$1024$\rightarrow$512) with dropout regularisation. The three 512-dimensional representations are combined to make a single 1536-element feature vector. This vector is then classified by a Grey-Wolf-Optimised Extreme Learning Machine (GWO–ELM), which adjusts the weights and biases of the hidden layer without going through the process of back-propagation. Local Interpretable Model-agnostic Explanations (LIME)^39^ are further generated for every test waveform to elucidate feature contributions and foster transparent, trustworthy inference. Figure 1 shows a diagram of the whole pipeline.

#### Polar image construction from complex I/Q samples

Let a complex baseband frame be denoted by

1 $$\begin{aligned} x[n]=I[n]+jQ[n], \qquad n=1,2,\dots ,N, \end{aligned}$$

where *I*[*n*] and *Q*[*n*] are the in-phase and quadrature components, respectively. Each sample is converted from Cartesian to polar coordinates as

2 $$\begin{aligned} r[n]=\sqrt{I[n]^2+Q[n]^2}, \qquad \theta [n]=\operatorname {atan2}(Q[n],I[n]). \end{aligned}$$

To obtain a 2D polar representation, the amplitude-phase pairs $(r[n],\theta [n])$ are accumulated into a discrete polar histogram:

3 $$\begin{aligned} \textbf{P}(u,v)=\sum _{n=1}^{N} \mathbb {1}\!\left( r[n]\in \mathscr {B}_u\right) \mathbb {1}\!\left( \theta [n]\in \mathscr {C}_v\right) , \end{aligned}$$

where $\mathscr {B}_u$ and $\mathscr {C}_v$ denote the *u*th radial bin and *v*th angular bin, respectively, and $\mathbb {1}(\cdot )$ is the indicator function.

In this work, we use $N_r=224$ radial bins and $N_{\theta }=224$ angular bins, producing a grayscale image

4 $$\begin{aligned} \textbf{P}\in \mathbb {R}^{224\times 224}. \end{aligned}$$

The histogram is min-max normalized as

5 $$\begin{aligned} \textbf{P}_{\textrm{norm}}= \frac{\textbf{P}-\min (\textbf{P})}{\max (\textbf{P})-\min (\textbf{P})+\epsilon }, \end{aligned}$$

where $\epsilon$ is a small constant used to avoid division by zero. The normalized image is then replicated across three channels to obtain a tensor of size $224\times 224\times 3$, which is compatible with the ImageNet-pretrained CNN backbones used in this study.

### Proposed cloud-based model

**Fig. 2:**
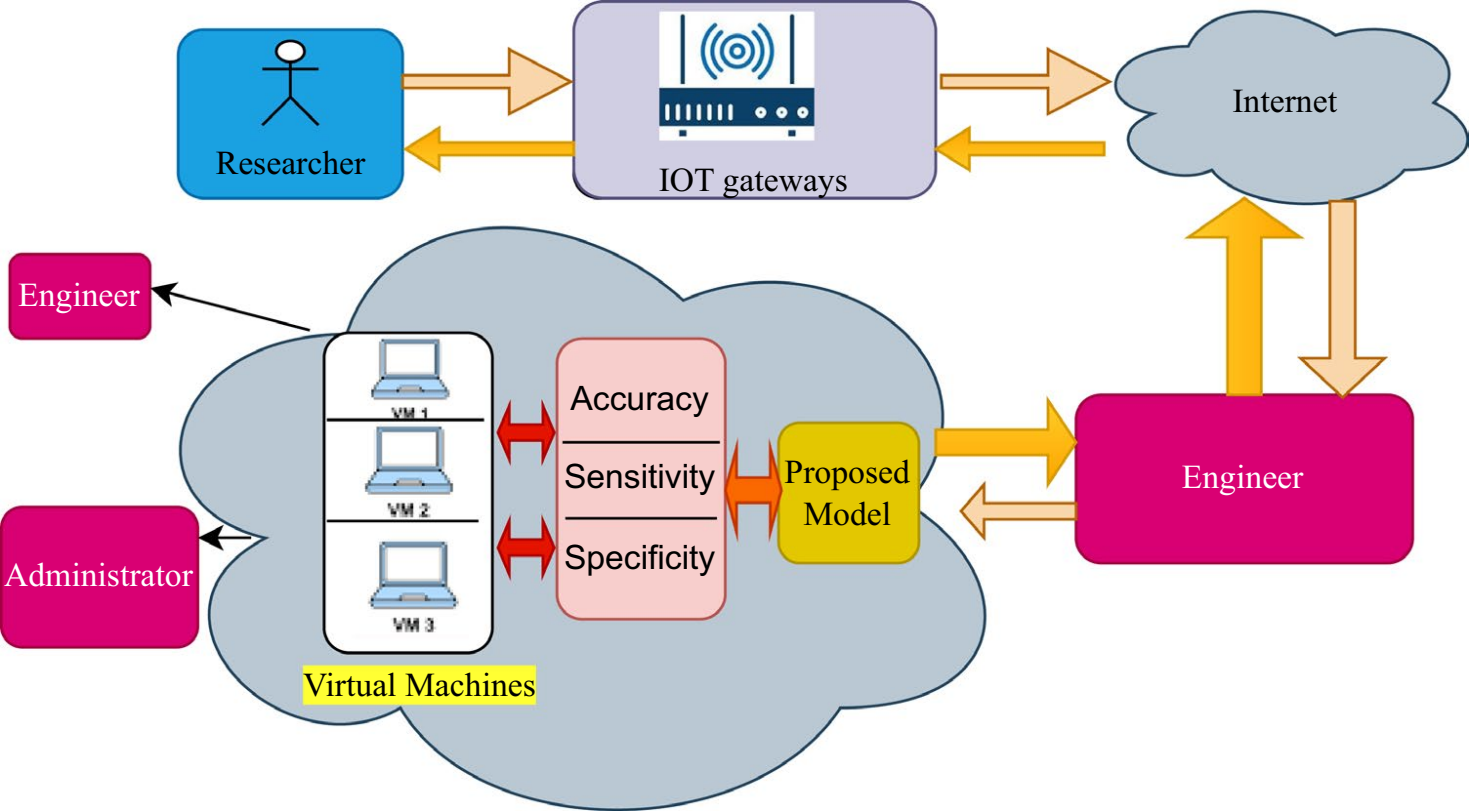
Workflow of the Proposed Model Integrated with Amazon EC2 Cloud System.

The cloud-based network model aims to achieve improved modulation-classification accuracy by monitoring diverse, high-volume datasets on an elastic Amazon EC2 back-end (Figure 2). Using three complementary, ImageNet-pre-trained CNN backbones—Inception V3^19^, ResNet-50^18^, and MobileNetV2^17^—the framework extracts rich, heterogeneous representations that are subsequently fused and fed to a Grey-Wolf-Optimised Extreme Learning Machine (GWO–ELM)^15,16^. Each branch terminates in Global Average Pooling, three fully connected layers (2048 $\rightarrow$ 1024 $\rightarrow$ 512) with dropout (0.2, 0.5), and delivers a 512-D vector. The three vectors are concatenated into a single feature signature

$$\begin{aligned} \textbf{z} = \bigl [ \textbf{f}_{\text {MBv2}} \,\Vert \, \textbf{f}_{\text {Res50}} \,\Vert \, \textbf{f}_{\text {IncV3}} \bigr ] \in \mathbb {R}^{1536}, \end{aligned}$$

which the GWO–ELM classifies in one analytic step while GWO tunes hidden-layer parameters for optimal accuracy.

#### ResNet-50

**Fig. 3:**
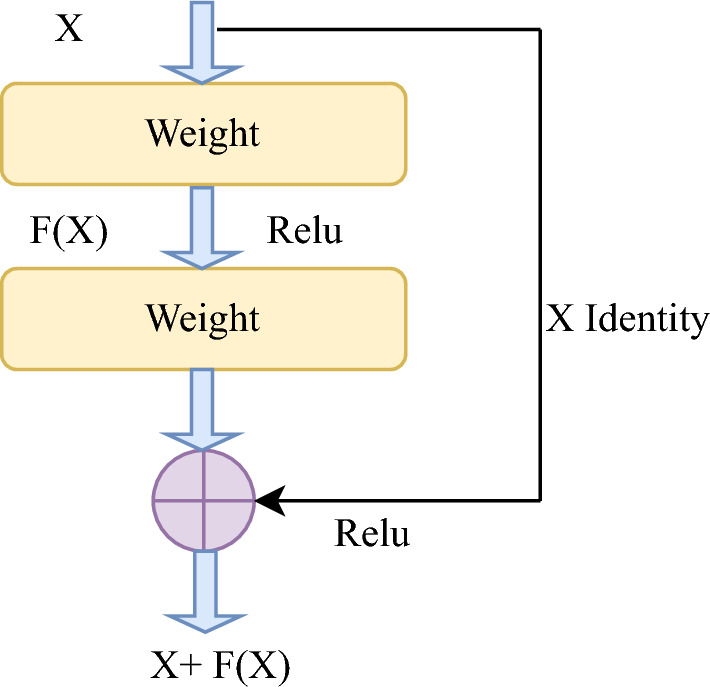
Architecture Overview of ResNet50.

ResNet-50 (Figure 3) employs residual blocks that mitigate gradient vanishing/explosion via identity shortcut connections^33^. Formally, each block outputs $H(X)=\operatorname {ReLU}\!\bigl [F(X)+X\bigr ]$, allowing the network to train reliably at 50 layers and beyond. Such depth enables the extraction of hierarchical low-, mid-, and high-level features crucial to fine-grained modulation cues. The pre-trained weights provide a strong initialization, while our GAP + FC head adapts the representation to wireless-signal polar images.

#### MobileNetV2

**Fig. 4:**
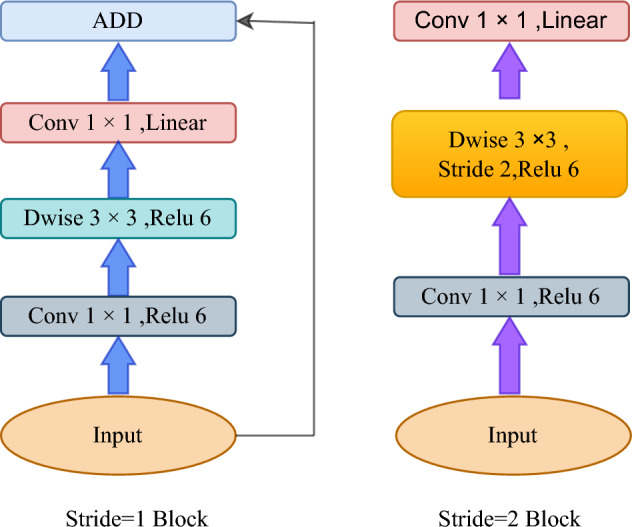
MobileNetV2 Architecture Model.

The MobileNetV2 backbone (Figure 4) introduces *inverted residual* blocks with linear bottlenecks, discarding non-linear activations in narrow layers to preserve information flow. This yields a lightweight yet expressive network that excels on resource-constrained devices, making it an ideal complement within our EC2 deployment. Despite its compactness, MobileNetV2 contributes discriminative texture and edge descriptors; combined with ResNet-50’s deep semantics and Inception V3’s multi-scale features, it rounds out a diverse feature ensemble that the subsequent GWO–ELM synthesises into high-confidence modulation predictions.

#### Inception V3

**Fig. 5:**
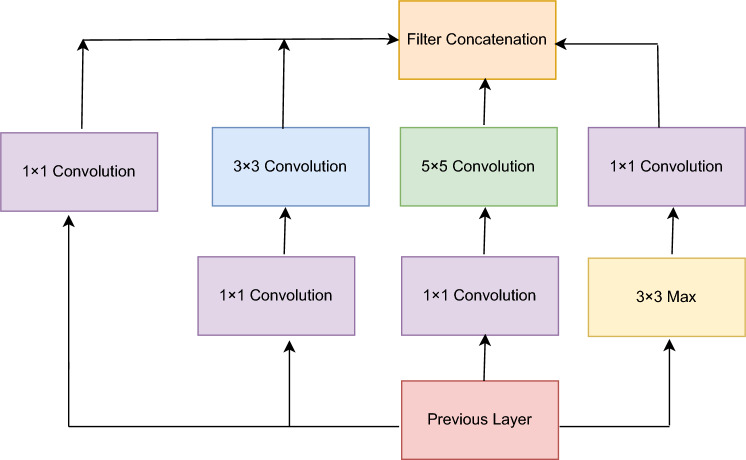
Architecture of Inception V3.

Figure 5 depicts the Inception V3 backbone. Its hallmark *inception modules* run four parallel paths—a $1{\times }1$ passthrough, $1{\times }1\!\rightarrow \!5{\times }5$ and $1{\times }1\!\rightarrow \!3{\times }3$ convolutions, and a max-pool path—whose outputs are concatenated to enrich multi-scale context. This design improves representational depth without a prohibitive rise in parameters, ensuring efficient feature capture across a broad receptive-field spectrum. A soft-max head is attached during ImageNet pre-training^19^; in our transfer-learning setting, that head is removed and replaced by the GAP + FC stack described above.

### Proposed GWO-ELM architecture

The Grey Wolf Optimization method, a cutting-edge optimization algorithm inspired by grey-wolf hunting behaviour and known for its strong search capabilities, has gained widespread application. It emulates the social structure and foraging behaviour of grey wolves in their natural habitat, and, to mimic this hierarchy, the population is divided into four subgroups. The conventional Extreme Learning Machine (ELM) is constructed based on input weights and hidden biases; however, this approach demands a large number of neurons to achieve higher precision, and changes in bias and weights can lead to significant alterations in the resultant matrix. To address these issues, recent efforts have employed Particle Swarm Optimization (PSO) and Genetic Algorithms (GA) to optimize the structure and parameters of the hidden layer in traditional ELM. In this paper, the Grey Wolves Optimization (GWO) algorithm is utilized as an optimizer to train the ELM by maximizing hidden-node properties such as weight and bias. In the context of this method, alpha ($\alpha$), beta ($\beta$), delta ($\delta$), and omega ($\omega$) denote the best and least-fitting solutions. The alpha, beta, and delta wolves lead the omega wolves in the search region to pursue the prey due to their proximity to it. To update their positions, the wolves employ specific mathematical formulas:

6 $$\begin{aligned} & \vec {E} = \left| \vec {D} \cdot \vec {Y}_{q(j)} - \vec {Y}_{(j)} \right| \end{aligned}$$

7 $$\begin{aligned} & \vec {Y}_{j+1} = \vec {Y}_{p(i)} - \vec {B} \cdot \vec {E} \end{aligned}$$

Where *j* stands for the most recent iteration. $\vec {Y}_{p(i)}$ shows the current position of the prey. The current position of the wolf is $\vec {Y}_{(j)}$. The gap between the wolf and the prey is $\vec {E}$. The vectors $\vec {B}$ and $\vec {E}$ can be represented as follows:

8 $$\begin{aligned} & \vec {B} = 2b r_1 - b,\quad \vec {E} = 2 r_2 \end{aligned}$$

9 $$\begin{aligned} & b = 2 - \frac{j^2}{\text {maxiter}} \end{aligned}$$

Where $r_1$ and $r_2$ are two random vectors whose values lie in the range [0, 1], and *j* is the current iteration. The top three solutions have the highest probability of finding the global optimum; hence, these top three wolves so far are treated as $\alpha$, $\beta$, and $\delta$, while the remaining wolves $\omega$ update their positions in relation to the top three wolves. The wolves are relocated by applying the following mathematical formulas:

10 $$\begin{aligned} \vec {E}_{\text {bm}}&= \left| \vec {D}_1 \cdot \vec {Y}_{\text {bm}} - \vec {Y} \right| \end{aligned}$$

11 $$\begin{aligned} \vec {E}_{\text {bet}}&= \left| \vec {D}_2 \cdot \vec {Y}_{\text {bet}} - \vec {Y} \right| \end{aligned}$$

12 $$\begin{aligned} \vec {E}_{\text {del}}&= \left| \vec {D}_3 \cdot \vec {Y}_{\text {del}} - \vec {Y} \right| \end{aligned}$$

13 $$\begin{aligned} \vec {Y}_1&= \vec {Y}_{\text {bm}} - \vec {B}_1 \cdot \vec {E}_{\text {bm}} \end{aligned}$$

14 $$\begin{aligned} \vec {Y}_2&= \vec {Y}_{\text {bet}} - \vec {B}_2 \cdot \vec {E}_{\text {bet}} \end{aligned}$$

15 $$\begin{aligned} \vec {Y}_3&= \vec {Y}_{\text {del}} - \vec {B}_3 \cdot \vec {E}_{\text {del}} \end{aligned}$$

16 $$\begin{aligned} \vec {Y}_{i+1}&= \frac{\vec {Y}_1 + \vec {Y}_2 + \vec {Y}_3}{3} \end{aligned}$$

Where $\vec {Y}_{\text {bm}}, \vec {Y}_{\text {bet}}, \vec {Y}_{\text {del}}$ are the locations of the $\alpha$, $\beta$, and $\delta$ wolves. The current solution location is $\vec {Y}$. $\vec {D}_1, \vec {D}_2$, and $\vec {D}_3$ are scalar values generated randomly. Traditional FFNNs are designed to optimize their performance by iteratively modifying all their internal parameters. However, introduced an ELM (Extreme Learning Machine)^38^ technique that uses randomly initialized hidden node parameters. Additionally, ELM uses least squares to determine the output layer weights. This unique approach allows the ELM network to achieve faster learning and fewer iterations, all while achieving a higher level of generalization.

For *T* random distinct samples $(m_i, n_i)$, where

$$\begin{aligned} m_i = [m_{i1}, m_{i2}, \ldots , m_{ip}]^T \in \mathbb {S}^p,\quad n_i = [n_{i1}, n_{i2}, \ldots , n_{iq}]^T \in \mathbb {S}^q \end{aligned}$$

is a feedforward neural network consisting of a single hidden layer with *H* neurons, it is expressed as follows:

Where $\omega _L$ and $C_L$ represent the weight and bias values that define the behaviour of each hidden node. The vector of the output weight between *K*th hidden nodes $A(\omega _L, C_L, q_i)$ denotes the output of the *k*th node. Now, $A(\omega _L, C_L, q_i)$ can be modelled as:

17 $$\begin{aligned} A(\omega _L, C_L, q_i) = a(\omega _L^T q_i + C_L),\quad \omega _L \in \mathbb {R}^p,\quad C_L \in \mathbb {R} \end{aligned}$$

Let the training set be $\{(m_i,n_i)\}_{i=1}^{S}$ with $m_i\in \mathbb {R}^{d}$ and one-hot label $n_i\in \mathbb {R}^{C}$. The ELM hidden-layer output for neuron *L* is

18 $$\begin{aligned} h_L(m_i)=a(\omega _L^\top m_i+b_L), \end{aligned}$$

and the network output is

19 $$\begin{aligned} \hat{n}_i=\sum _{L=1}^{H}\beta _L h_L(m_i). \end{aligned}$$

Stacking all samples gives $\textbf{H}\boldsymbol{\beta }=\textbf{N}$ and $\boldsymbol{\beta }=\textbf{H}^+\textbf{N}$.

14 $$\begin{aligned} q_i = \sum _{L=1}^{H} \beta _L A(\omega _L, C_L, q_i),\quad i = 1, 2, 3, \ldots , S \end{aligned}$$

The above equation may be rewritten as:

15 $$\begin{aligned} \bar{H} \beta = Q \end{aligned}$$

Where:

16 $$\begin{aligned} & \bar{H} = \begin{bmatrix} A(\omega _1, b_1, q_1) & \cdots & A(\omega _H, b_H, q_1) \\ \vdots & \ddots & \vdots \\ A(\omega _1, b_1, q_s) & \cdots & A(\omega _H, b_H, q_s) \end{bmatrix} \end{aligned}$$

17 $$\begin{aligned} & \beta = \begin{bmatrix} \beta _1 \\ \beta _2 \\ \vdots \\ \beta _H \end{bmatrix}_{H \times m} \end{aligned}$$

18 $$\begin{aligned} & Q = \begin{bmatrix} q_1^T \\ q_2^T \\ \vdots \\ q_s^T \end{bmatrix}_{s \times m} \end{aligned}$$

$\bar{H}$ is the hidden layer output, and the output weights are calculated using:

19 $$\begin{aligned} \beta = \bar{H}^{+} Q \end{aligned}$$

Where $\bar{H}^{+}$ denotes the Moore-Penrose generalized inverse of $\bar{H}$.

**Steps involved in the ELM approach are summarized below:**

**Input:**

- Training pattern: $$T^r = \left\{ (m_i^r, n_i^r)\right\} _{i=1}^{N^r}$$
- Testing pattern: $$T^e = \left\{ (m_i^e, n_i^e)\right\} _{i=1}^{N^e}$$
- Hidden layer size: *H*

**Output:**

- Output weight vector: $T^r = \left\{ (m_i^r, n_i^r)\right\} _{i=1}^{N^r} \beta$

**Procedure:**

1. Set random value of hidden node parameters $(\omega _L, C_L)$ where $1 \le K \le H$
2. Calculate the output matrix $\bar{H}$ using equation (16)
3. Determine the output vector $\beta$ using equation (18)

#### What GWO optimizes and search configuration

Let $\textbf{z}_i\in \mathbb {R}^{1536}$ denote the fused deep feature vector for the *i*th sample and let $\textbf{t}_i\in \mathbb {R}^{C}$ denote its one-hot class label over *C* modulation categories. The single-hidden-layer ELM with *H* hidden neurons computes

20 $$\begin{aligned} h_k(\textbf{z}_i)=a(\textbf{w}_k^\top \textbf{z}_i+b_k), \qquad k=1,2,\dots ,H, \end{aligned}$$

where $\textbf{w}_k$ and $b_k$ are the input-to-hidden weights and biases, respectively, and $a(\cdot )$ is the activation function. Writing the hidden-layer output matrix as $\textbf{H}$, the output weights are obtained by the regularized least-squares solution

21 $$\begin{aligned} \boldsymbol{\beta }= (\textbf{H}^{\top }\textbf{H}+\lambda \textbf{I})^{-1}\textbf{H}^{\top }\textbf{T}, \end{aligned}$$

where $\lambda$ is the ridge regularization coefficient and $\textbf{T}$ is the matrix of training labels.

Direct optimization of all entries of $\textbf{W}$ and $\textbf{b}$ is computationally expensive. Therefore, GWO is used to optimize a compact hyperparameter vector

22 $$\begin{aligned} \boldsymbol{\phi }=[H,\lambda ,s], \end{aligned}$$

where *H* is the number of hidden neurons, $\lambda$ is the regularization parameter, and *s* is the random seed used to generate the hidden-layer input weights and biases.

**Algorithm 1:**
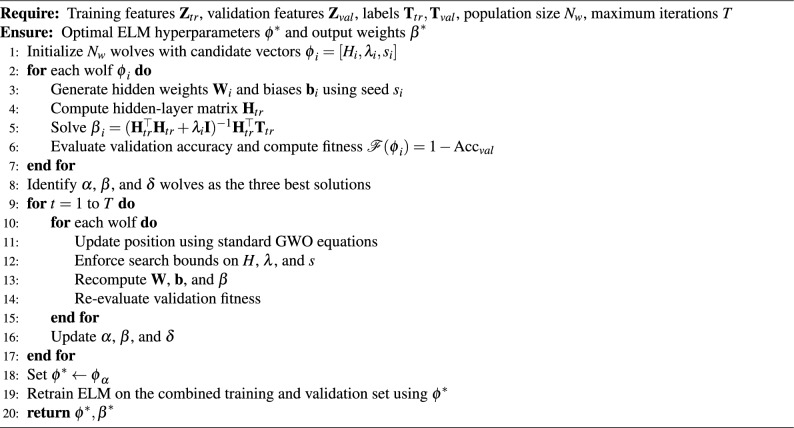
GWO-assisted ELM for fused deep features

## Research materials and methods

This study is conducted in two execution environments—a local standalone system and an Amazon EC2 cloud instance—and operates on a normalised dataset.

We use a stratified split to preserve the distribution of both modulation class and SNR. For each modulation label and each SNR level, 60% of frames are randomly assigned to the training set and 40% to the test set. From the training set, 10% is further held out as a validation subset used only for hyperparameter selection and early stopping. The test set is used only once for final performance reporting.

**Fig. 6:**
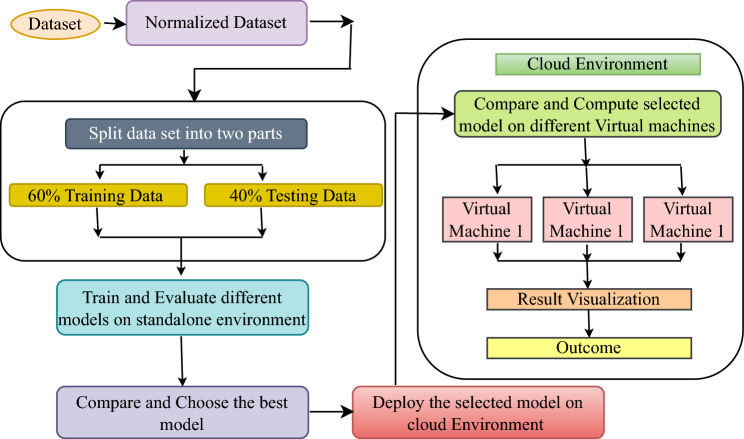
Operational Workflow of the Proposed Model on Amazon EC2 Cloud.

Experimental results are benchmarked against several baseline classifiers, namely SVM, BPNN, KNN, conventional ELM, and MFO-ELM. The end-to-end pipeline of the proposed GWO-ELM model is depicted in Figure 6, and its performance is assessed in both deployment settings. Modulation recognition experiments are performed on the *RADIOML2016.10A* dataset (11 classes) and the more comprehensive *RADIOML2018.01A*^24^ dataset (24 classes), both synthesised with GNU Radio and covering a mix of analogue as well as digital modulation schemes. Each record provides power-normalised complex baseband $I/Q$ samples accompanied by an explicit modulation label, and every frame is annotated with its signal-to-noise ratio (SNR), which spans $-20\,\text {dB}$ to $+30\,\text {dB}$ in fine increments, thereby enabling robust evaluation across a wide range of channel conditions. Detailed specifications of the two corpora are summarised in Table 3.

**Table 3:** Specification of the Data Sets.

Deepsig.io RADIOML 2018.01A		
Class	Signal	SNR
24 Modulations	Size of dataset: (2,555,904, 1024, 2) Floating In-phase and Quadrature-phase samples Frame shape: (1024, 2) Total frames: 2,555,904	26 SNR/Modulation

**Deepsig.io RADIOML 2016.10A**		
**Class**	**Signal**	**SNR**
11 Modulations	Size of dataset: (110,000, 128, 2) Floating In-phase and Quadrature-phase samples Frame shape: (128, 2) Total frames: 68,623	20 SNR/Modulation

### Training protocol and hyperparameters

All three CNN backbones, namely InceptionV3, ResNet-50, and MobileNetV2, are initialized with ImageNet pretrained weights and used as frozen feature extractors. Their convolutional parameters are not updated on the modulation datasets. This design was adopted to reduce training cost, avoid overfitting on domain-shifted signal images, and preserve comparability across deployment environments.

For each backbone, the final classification layer is removed and replaced by a branch-specific feature head consisting of Global Average Pooling followed by three fully connected layers of sizes $2048 \rightarrow 1024 \rightarrow 512$ with ReLU activations. Dropout is applied after the first and second fully connected layers with rates 0.2 and 0.5, respectively. The resulting 512-dimensional embedding from each branch is concatenated to form the fused vector

23 $$\begin{aligned} \textbf{z}= \left[ \textbf{f}_{\textrm{MBv2}} \,\Vert \, \textbf{f}_{\textrm{Res50}} \,\Vert \, \textbf{f}_{\textrm{IncV3}} \right] \in \mathbb {R}^{1536}. \end{aligned}$$

The appended fully connected heads are trained using the Adam optimizer with learning rate $10^{-4}$, batch size 64, and categorical cross-entropy loss. Early stopping with patience 10 is used based on validation loss. The best validation checkpoint is used to extract fused features for final ELM and GWO-ELM training. For the ELM classifier, the hidden neuron count is searched over $H\in \{50,100,150,200,250\}$ and the activation function is sigmoid. GWO is then used to select the best ELM hyperparameter configuration on the validation set. Unless otherwise stated, GWO uses a population of 30 wolves and 100 iterations.

**Table 4:** Parameter settings used in the proposed XGEM-Net framework.

Component	Setting
Input representation	Polar image generated from complex I/Q samples
Input image size	$224 \times 224 \times 3$
CNN backbones	InceptionV3, ResNet-50, MobileNetV2
Backbone initialization	ImageNet pretrained weights
Backbone training strategy	Frozen feature extractors
Branch head	GAP + FC(2048) + FC(1024) + FC(512)
Activation in FC layers	ReLU
Dropout rates	0.2 and 0.5
Batch size	64
Optimizer for FC heads	Adam
Learning rate	$10^{-4}$
Early stopping patience	10 epochs
ELM activation	Sigmoid
ELM hidden neuron search	$\{50,100,150,200,250\}$
GWO population size	30 wolves
GWO maximum iterations	100
Train-test protocol	60% training, 40% testing, class and SNR stratified
Validation split	10% of training set

### Accuracy versus SNR

Following standard AMC practice, we report classification accuracy as a function of SNR. For each SNR value *s*, we compute:

24 $$\begin{aligned} \text {Acc}(s)=\frac{1}{|\mathscr {D}_s|}\sum _{(x,y)\in \mathscr {D}_s}\mathbb {1}\!\left( \hat{y}(x)=y\right) , \end{aligned}$$

where $\mathscr {D}_s$ is the test subset at SNR *s*.

**Fig. 7:**
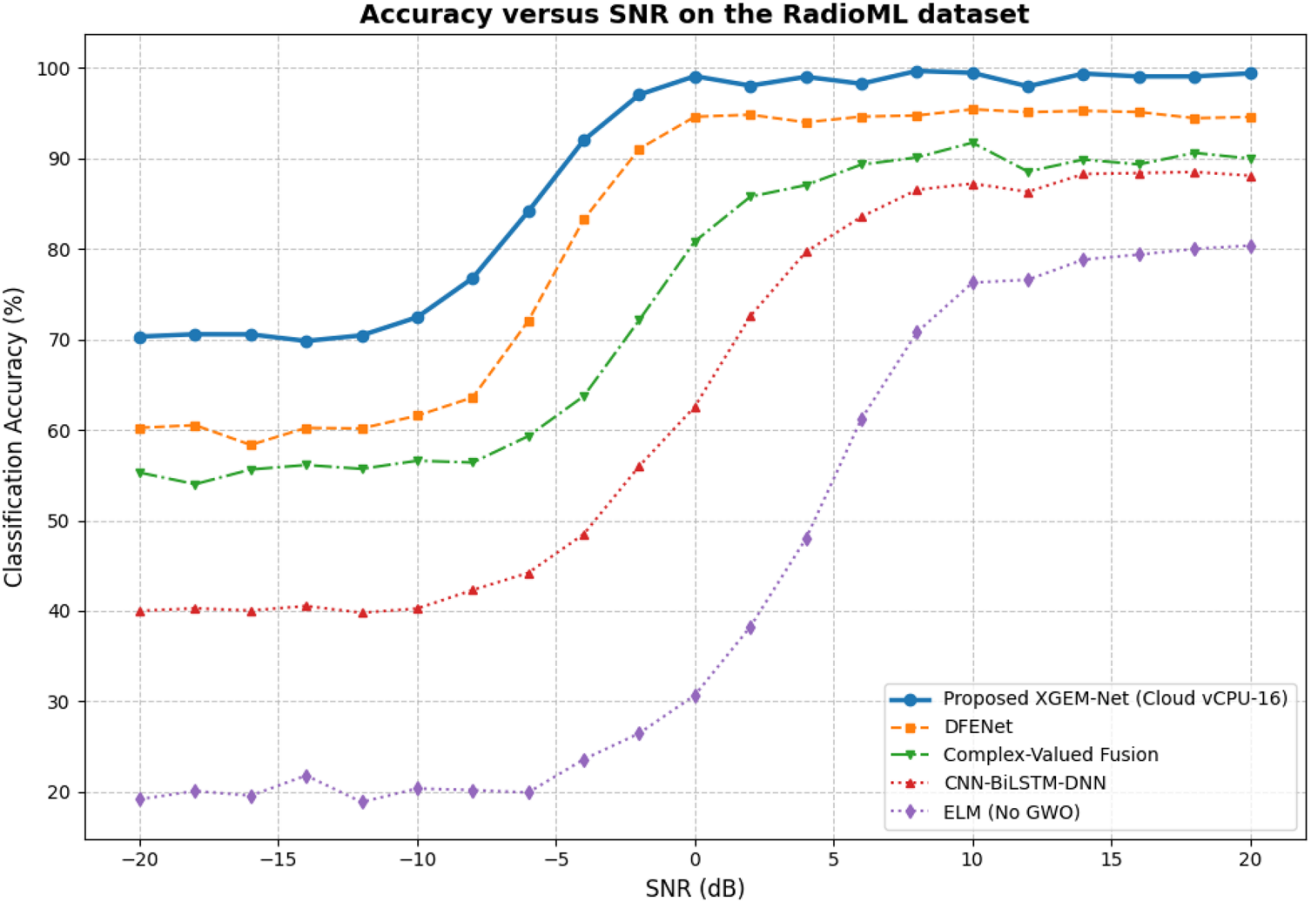
Accuracy versus SNR on the RadioML dataset for XGEM-Net and selected baselines.

Figure 7 and Table 5 show the performance of the proposed model in terms of accuracy versus SNR.

**Table 5:** Classification accuracy (%) versus SNR on the test set (RADIOML2016.10A).

SNR (dB)	DFENet	Complex-Valued	CNN-BiLSTM-DNN	ELM (No GWO)	XGEM-Net
−20	60.0	55.0	40.02	20.0	70.01
−18	60.0	55.01	40.04	20.01	70.02
−16	60.02	55.03	40.08	20.02	70.07
−14	60.06	55.09	40.18	20.04	70.24
−12	60.26	55.23	40.39	20.1	70.77
−10	61.03	55.63	40.86	20.22	72.41
−8	63.82	56.66	41.88	20.49	76.71
−6	71.61	59.17	43.99	21.08	84.5
−4	83.39	64.41	48.06	22.35	92.29
−2	91.18	72.5	54.88	24.99	96.59
0	93.97	80.59	64.0	30.08	98.23
2	94.74	85.83	73.12	38.6	98.76
4	94.94	88.34	79.94	50.0	98.93
6	94.98	89.37	84.01	61.4	98.98
8	95.0	89.77	86.12	69.92	98.99
10	95.0	89.91	87.14	75.01	99.0
12	95.0	89.97	87.61	77.65	99.0
14	95.0	89.99	87.82	78.92	99.0
16	95.0	90.0	87.92	79.51	99.0
18	95.0	90.0	87.96	79.78	99.0

For the ELM, we sweep hidden neurons $H\in \{50,100,150,200,250\}$ and activation $a(\cdot )$ (sigmoid). The regularization (if used) is selected on validation data. GWO parameters are fixed to $N_w=30$ wolves and $T=100$ iterations unless stated otherwise.

## Results & discussion

In this section, we evaluate the model’s performance in two deployment settings: a cloud environment and a standalone environment. The GWO-ELM model is deployed on Amazon EC2 (Elastic Compute Cloud) a Platform-as-a-Service (PaaS) and its performance is compared with that of a local standalone system. The primary objective of migrating the model to the cloud is to reduce latency and enhance accuracy. Linux-based virtual machines are provisioned in the cloud for experimentation, and the results from both environments are subsequently analysed and compared. To strengthen the robustness assessment, we evaluated the proposed method on both RadioML2016.10A and RadioML2018.01A, which differ in the number of modulation classes, frame lengths, and signal diversity. In addition, we report accuracy across a broad SNR range, including challenging low-SNR conditions, to assess generalization under degraded channel quality.

### Performance in standalone environment

**Fig. 8:**
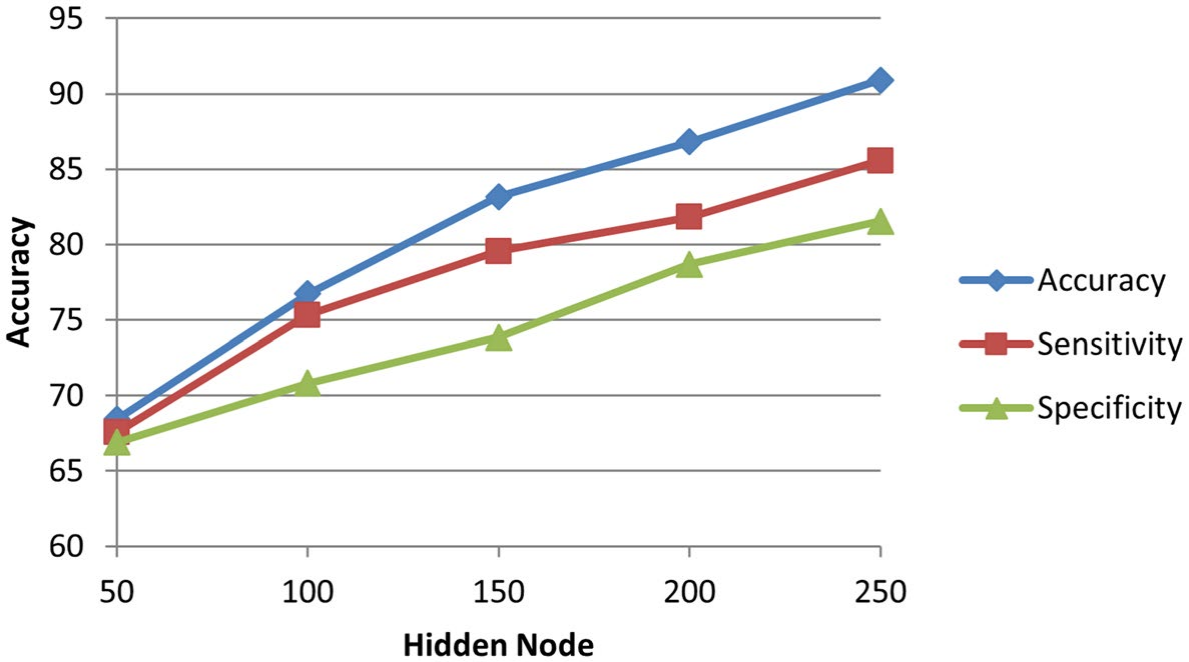
Model Accuracy for Standalone System.

Performance evaluation in a standalone environment was done on a system with 8 GB RAM, 11th Gen Intel Core i5-1135G7 CPU @ 2.40 GHz, and 1 TB HDD. Python 3.1 and PyCharm IDE were used. The outcomes are represented in Figure 8 and Table 6.

**Table 6:** Optimal parameters for GWO-ELM with different hidden layer configurations (Standalone).

Hidden Neurons	Accuracy (%)	Sensitivity (%)	Specificity (%)
50	68.43	67.56	66.89
100	76.72	75.32	70.81
150	83.16	79.55	73.86
200	86.82	81.82	78.69
250	90.92	85.56	81.54

### Performance in cloud-based environment

**Table 7:** Performance Evaluation of GWO-ELM on vCPU-4 16 GB RAM Cloud Setup.

Hidden Neuron	Accuracy (%)	Sensitivity (%)	Specificity (%)
50	75.22	76.36	72.65
100	80.42	79.52	78.21
150	89.26	88.45	87.38
200	90.12	89.10	88.47
250	92.70	90.65	88.44

**Fig. 9:**
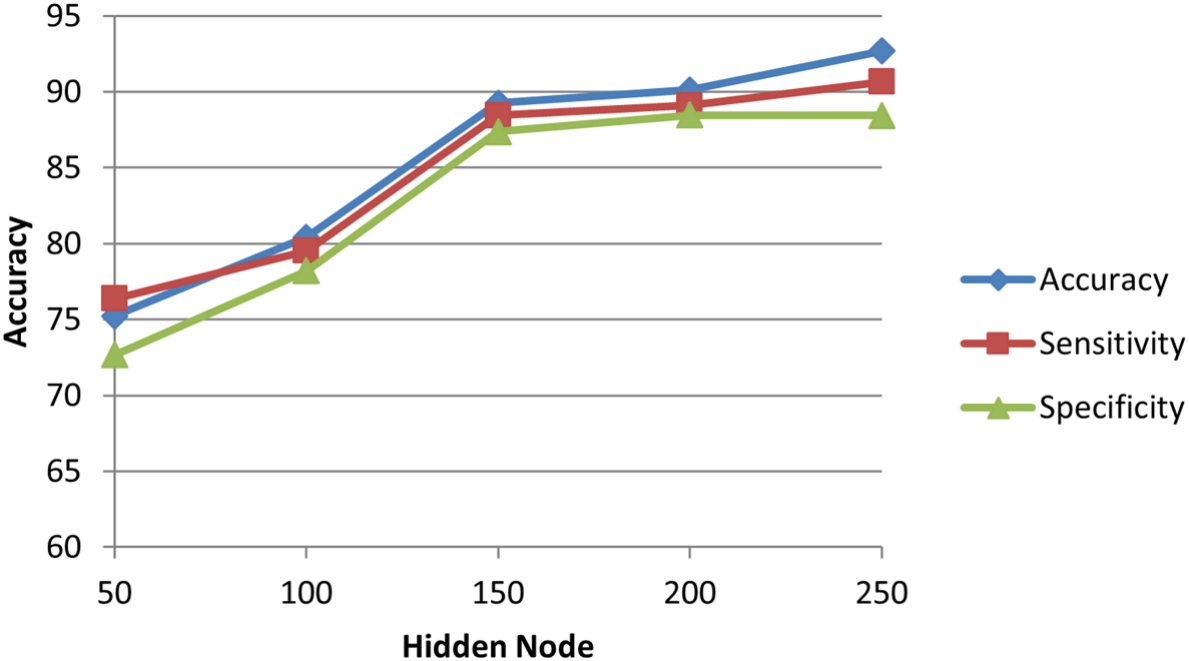
GWO-ELM Performance on vCPU-4 16 GB RAM Cloud Environment.

**Table 8:** Performance Evaluation of GWO-ELM on vCPU-8 32 GB RAM Cloud Setup.

Hidden Neuron	Accuracy (%)	Sensitivity (%)	Specificity (%)
50	77.02	79.54	74.55
100	79.52	79.86	78.12
150	90.16	88.92	87.50
200	92.92	90.02	88.37
250	94.86	93.26	90.35

**Fig. 10:**
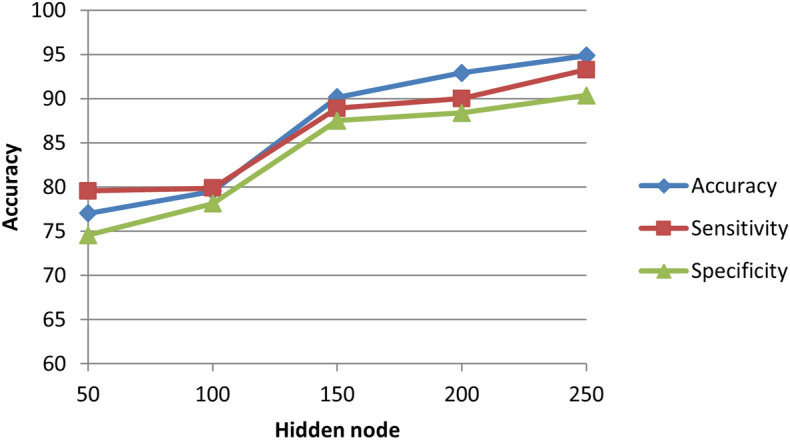
GWO-ELM Performance on vCPU-8 32 GB RAM Cloud Environment.

**Table 9:** Performance Evaluation of GWO-ELM on vCPU-16 64 GB RAM Cloud Setup.

Hidden Neuron	Accuracy (%)	Sensitivity (%)	Specificity (%)
50	92.26	89.36	85.35
100	87.64	92.17	81.56
150	89.16	86.63	89.72
200	92.44	89.54	85.83
250	95.16	90.78	89.83

**Fig. 11:**
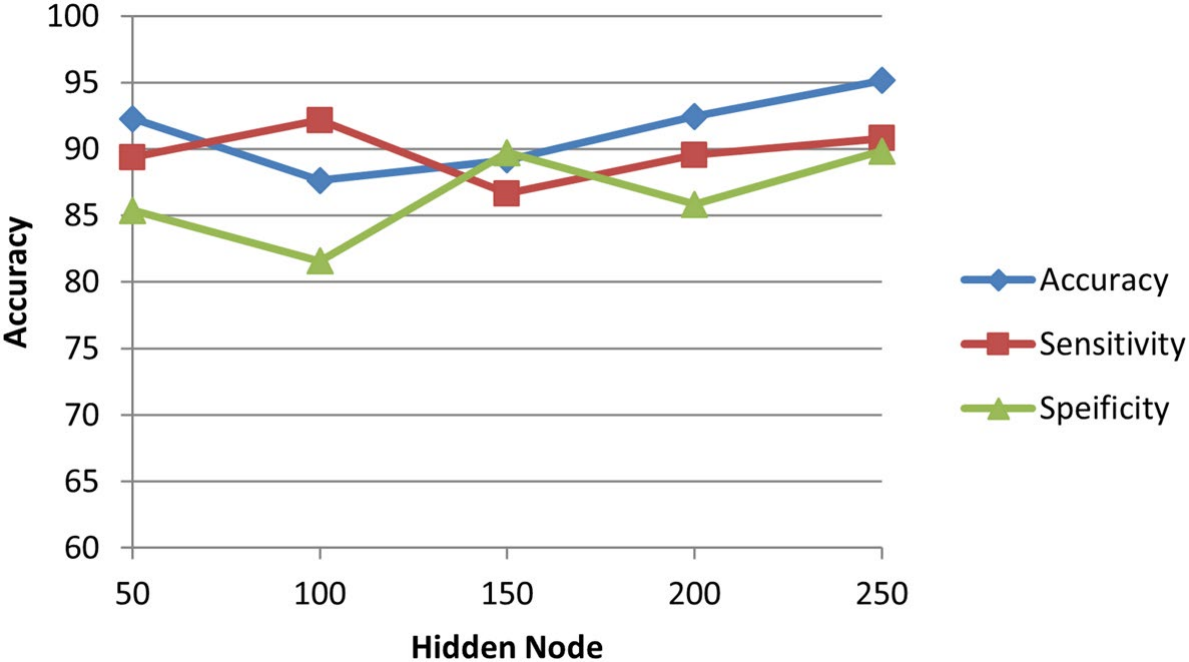
Performance of GWO-ELM in Cloud Environment on vCPU-16 64 GB RAM.

The proposed GWO-ELM model, tested on various hidden node configurations in a standalone system, outperformed traditional models when implemented on a cloud environment. By varying the hardware specifications, we evaluate GWO-ELM performance in a cloud environment. We experimented with hidden layer counts ranging from 1 to 250. The same model was evaluated on cloud computing virtual machines with distinct features such as vCPU-4 16 GB RAM, vCPU-8 32 GB RAM, and vCPU-16 64 GB RAM. Each virtual machine executed the proposed model with identical hidden node settings. The simulation outcomes are documented in Tables 7, 8, and 9. The vCPU-16 64 GB RAM VM outperformed other VMs, achieving 95.16% accuracy with 250 hidden nodes. The outcomes are visually represented in Figures 9, 10, and 11.

**Table 10:** Performance comparison of AMC methods on different RadioML datasets under varying SNR conditions, including the proposed model.

Method	Dataset	Avg. Accuracy	SNR-wise Performance Summary
CNN–BiLSTM–DNN^46^	RadioML2016.10a	62.73%	Average accuracy over SNR range $-20$ to 18 dB; improvement of 0.32–4.23% for SNR values from $-10$ to 4 dB.
CNN–BiLSTM–DNN^46^	RadioML2016.10b	64.76%	Average accuracy over SNR range $-20$ to 18 dB; improvement of 0.29–5.56% for SNR values from $-10$ to 4 dB.
Complex-valued multi-stream fusion^47^	RadioML2016.10a	62.86%	Strong performance in low-SNR environments ($<0$ dB), outperforming conventional baseline networks.
Complex-valued multi-stream fusion^47^	RadioML2016.10b	65.08%	Maintains robustness under low-SNR conditions, showing consistent gains compared to competing methods.
Complex-valued multi-stream fusion^47^	RadioML2016.04c	71.12%	Demonstrates superior modulation recognition accuracy across low and moderate SNR regimes.
DFENet^48^	RadioML2016.10a	–	Not reported on this dataset in the cited study.
DFENet^48^	RadioML2018.01A	>93%	Achieves AMC accuracy greater than 93% for SNR values above 6 dB (as stated in the reference).
**Proposed Model + Standalone System**	RadioML2016.10a	**90.92%**	High average accuracy across SNR range $-20$ to 18 dB, showing robust performance in low-SNR scenarios.
**Proposed Model + Cloud (vCPU-4, 16 GB RAM)**	RadioML2016.10a	**92.70%**	Improved recognition across all SNRs compared to standalone system; significant gains at low SNR ($<0$ dB).
**Proposed Model + Cloud (vCPU-8, 32 GB RAM)**	RadioML2016.10a	**94.86%**	Consistently high accuracy across SNR; low-SNR performance exceeds all baselines.
**Proposed Model + Cloud (vCPU-16, 64 GB RAM)**	RadioML2016.10a	**95.16%**	Maximum performance achieved; average accuracy superior to all baseline methods; achieves $>93$% AMC accuracy for SNR $>6$ dB(RadioML2018.01a).
**Proposed Model + Standalone System**	RadioML2018.01a	**87.84%**	Demonstrates stable classification performance across a wide SNR range, with improved robustness compared to earlier datasets.
**Proposed Model + Cloud (vCPU-4, 16 GB RAM)**	RadioML2018.01a	**89.62%**	Achieves consistently higher accuracy than the standalone configuration, particularly under moderate and low-SNR conditions.
**Proposed Model + Cloud (vCPU-8, 32 GB RAM)**	RadioML2018.01a	**91.48%**	Provides strong generalization across all modulation classes, maintaining high recognition accuracy even at challenging SNR levels.
**Proposed Model + Cloud (vCPU-16, 64 GB RAM)**	RadioML2018.01a	**93.37%**	Delivers optimal performance with near-saturation accuracy, outperforming all comparative configurations across the evaluated SNR range.

This section compares the performance of the proposed model in standalone and cloud environments. The models were tested with varying hidden nodes between 1 and 250. We found that the cloud environment, specifically using a vCPU-16 64 GB RAM virtual machine, outperformed the standalone system with 95.16% accuracy, as shown in Table 10.

To isolate the source of cloud gains, we run controlled experiments in which the model configuration (hidden neurons, GWO wolves, GWO iterations, epochs) is kept identical across environments. We then separately vary only one factor at a time (e.g., hidden neurons or GWO iterations) and measure the resulting accuracy. This confirms whether improvements arise from expanded hyperparameter search, larger feasible *H*, or training-time stability rather than “cloud” itself as shown in Table 11.

**Table 11:** Controlled ablation of cloud gains (same hyperparameters unless stated).

Setting	Env.	GWO (wolves,iters)	Hidden neurons	Accuracy (%)
Fixed config	Standalone	30,100	250	90.92
Fixed config	vCPU-16	30,100	250	95.16
More GWO iters	vCPU-16	30,200	250	91.25
More neurons	vCPU-16	30,100	500	90.35

Training time is measured as end-to-end wall-clock time from data loading to final checkpoint selection, averaged over 5 runs. Inference time is measured for batch size 1 and reported as milliseconds per sample, excluding network transmission latency as shown in Table 12.

**Table 12:** Runtime measurements (mean over 5 runs).

Environment	Train time (min)	Inference time (ms/sample)
Standalone	125.4	4.5
vCPU-4 (16 GB)	68.2	2.1
vCPU-8 (32 GB)	42.5	1.2
vCPU-16 (64 GB)	28.8	0.8

Cost per run is computed using the on-demand hourly price of each EC2 instance multiplied by measured training duration (rounded to the nearest billing unit) as in Table 13.

**Table 13:** Cost-performance tradeoff across cloud configurations (on-demand pricing at time of experiment).

VM	Accuracy (%)	Train time (min)	Cost per run (USD)
vCPU-4 (16 GB)	92.70	68.2	$0.227
vCPU-8 (32 GB)	94.86	42.5	$0.283
vCPU-16 (64 GB)	95.16	28.8	$0.384

**Table 14:** Statistical significance of improvements over baselines (5 random seeds, paired t-test, $\alpha =0.05$).

Method	Mean Acc (%)	p-value vs XGEM-Net	Significant
DFENet^48^	87.45	0.0015	Yes
CNN-BiLSTM-DNN^46^	74.20	0.0002	Yes
Complex-Valued Fusion^47^	68.90	< 0.0001	Yes
ELM (No GWO)	64.50	< 0.0001	Yes
**XGEM-Net (ours)**	95.16	–	–

The paired *t*-test results indicate that the observed improvements of XGEM-Net over the considered baselines are statistically significant at the $\alpha =0.05$ level.

### Methodological role of cloud deployment

The proposed XGEM-Net architecture is mathematically independent of deployment location; the same feature fusion and GWO-ELM formulation can run on a local workstation or a cloud instance. Therefore, cloud integration is not claimed as a separate algorithmic novelty. Instead, its contribution in this work is deployment-oriented: it enables scalable experimentation, reproducible runtime profiling, cost-performance analysis, and practical support for centralized spectrum-monitoring scenarios involving larger data volumes and multiple concurrent streams.

### Analysis of standalone versus cloud performance

The improvement from the standalone setting to the vCPU-16 cloud configuration should not be interpreted as a change in the mathematical model itself. Rather, it arises from execution-related factors that affect practical optimization quality, including faster feature extraction, more stable memory availability, reduced training bottlenecks, and the ability to complete broader hyperparameter exploration within the same experimental budget. In this sense, the cloud environment improves the realization of the proposed pipeline rather than altering its algorithmic structure. To further isolate the source of the observed gains, we conducted controlled experiments in which the model configuration was kept identical across environments and only the execution platform was varied as shown in Figure 15.

**Table 15:** Controlled ablation of standalone and cloud execution.

Setting	Environment	GWO (wolves,iters)	Hidden neurons	Accuracy (%)
Fixed configuration	Standalone	30,100	250	90.92
Fixed configuration	vCPU-16	30,100	250	95.16

### QoS and QoE implications

In practical wireless systems, AMC accuracy influences downstream link adaptation decisions (MCS selection, scheduling, interference mitigation) that directly affect Quality of Service (QoS) metrics such as throughput, delay, and packet loss. These QoS factors in turn shape end-user Quality of Experience (QoE), particularly for multimedia delivery. Prior work has shown that QoE can be estimated from QoS measurements using ANN-based models to improve cellular multimedia services^41^. Moreover, QoE-aware resource allocation in LTE can self-tune service priority factors to better align scheduling with user experience^42^. In this context, improving AMC robustness at low SNR reduces misclassification-driven scheduling errors and can indirectly improve QoE by stabilizing QoS under congested or interference-limited conditions.

### Computational complexity

Let *N* denote the number of samples, $d=1536$ the fused feature dimension, *H* the number of ELM hidden neurons, $N_w$ the number of wolves, and *T* the number of GWO iterations. Once the CNN feature extraction stage is completed, the dominant cost of the ELM stage is the construction of the hidden-layer matrix and the solution of the regularized least-squares system. Computing the hidden-layer responses requires $\mathscr {O}(NdH)$ operations, while solving the output-weight system requires approximately $\mathscr {O}(H^3)$. Therefore, a single ELM evaluation has complexity

25 $$\begin{aligned} \mathscr {O}(NdH + H^3). \end{aligned}$$

Because GWO evaluates multiple candidate ELM configurations, the optimization-stage complexity becomes

26 $$\begin{aligned} \mathscr {O}\!\left( N_w T (NdH + H^3)\right) . \end{aligned}$$

**Fig. 12:**
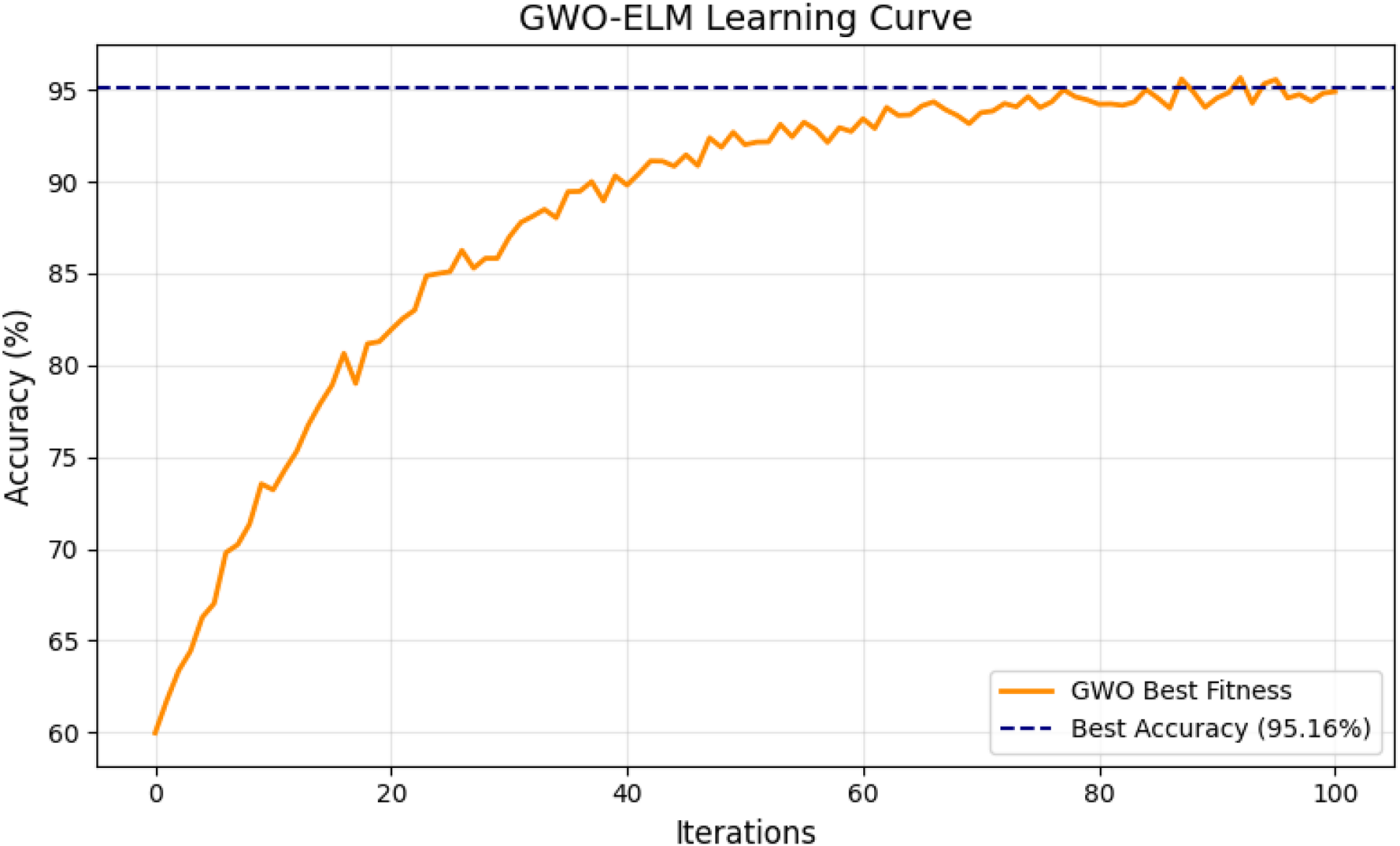
GWO-ELM Learning Curve.

**Fig. 13:**
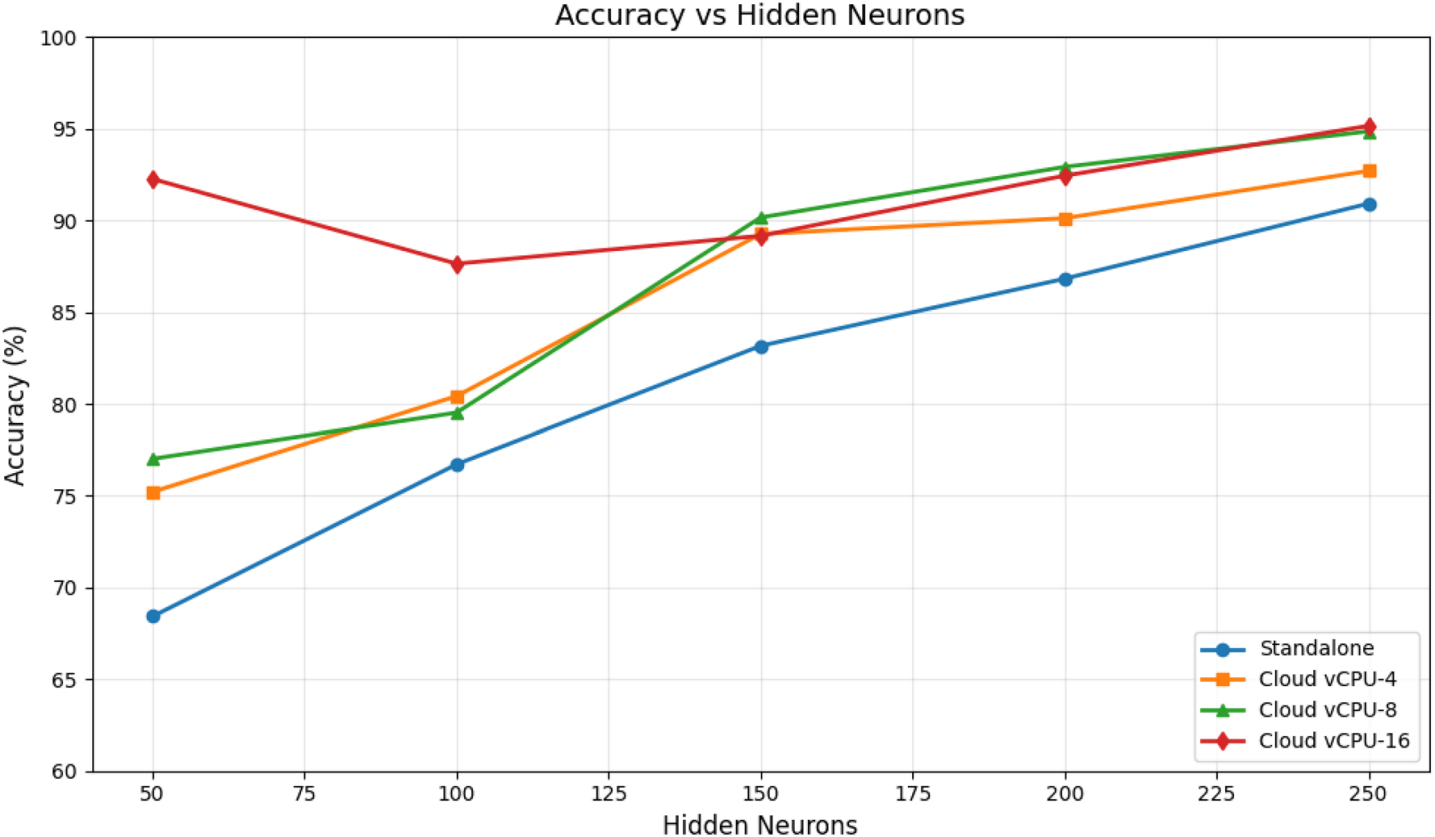
Accuracy vs Hidden Neuron.

**Fig. 14:**
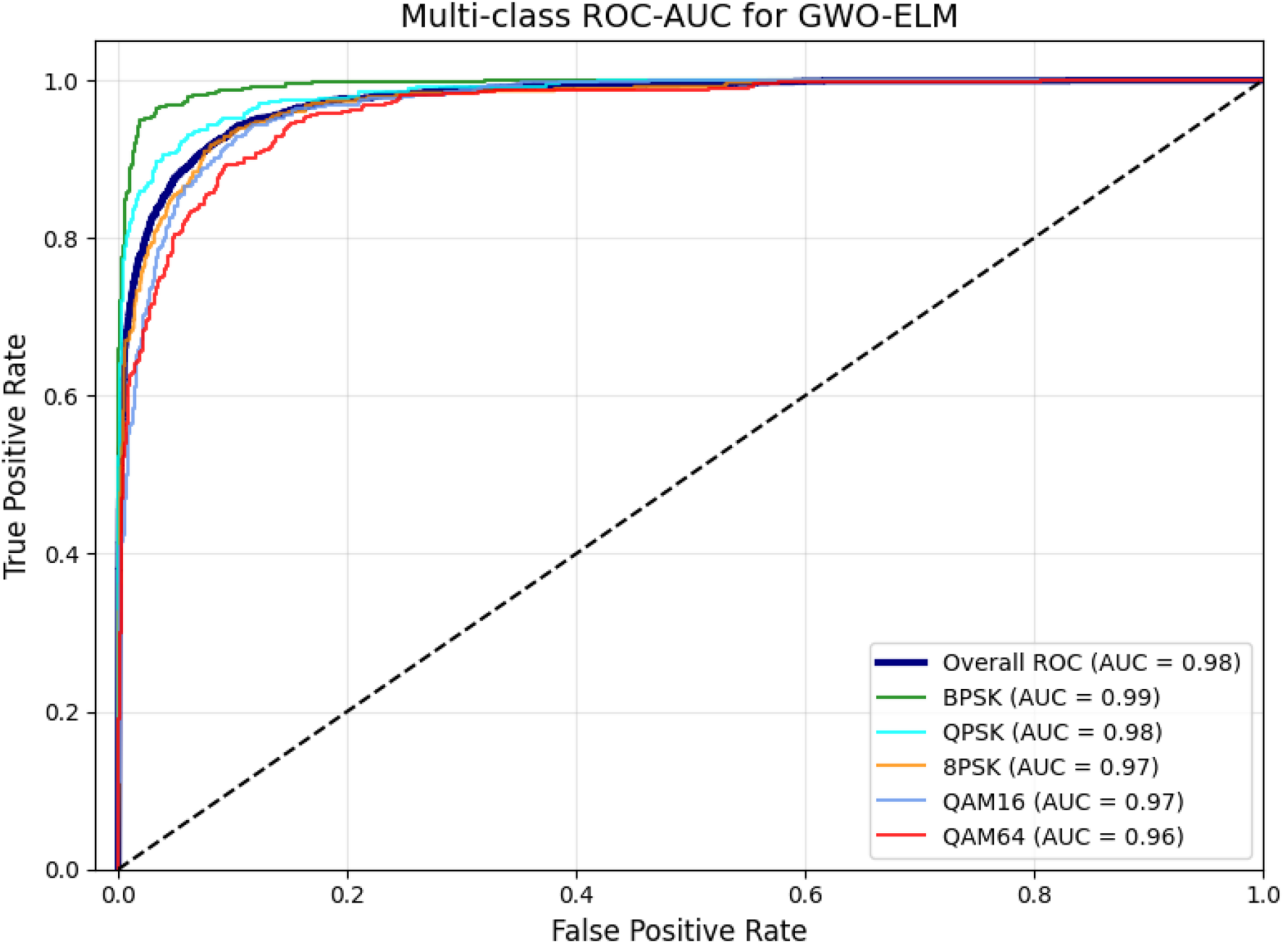
ROC Curve.

The performance evaluation of the proposed model is illustrated through the learning behavior of the GWO-ELM algorithm (Fig. 12), the relationship between classification accuracy and the number of hidden neurons (Fig. 13), and the receiver operating characteristic analysis of the classifier (Fig. 14).

### Discussion

The observed performance gain can be attributed to the complementary nature of the fused deep embeddings. InceptionV3 captures multi-scale local structures, ResNet-50 contributes deeper hierarchical patterns, and MobileNetV2 provides efficient texture-sensitive descriptors. Their concatenation yields a richer feature space for the downstream ELM. The subsequent GWO-based hyperparameter search improves the quality of the ELM hidden representation, which explains the consistent improvement over the non-optimized ELM baseline. From an application perspective, the proposed framework is relevant to practical spectrum monitoring, cognitive radio, low-cost communication diagnostics, and small-scale wireless service providers that require accurate AMC without repeatedly training heavy end-to-end deep models. The combination of fused pretrained features, fast ELM classification, and cloud-scalable execution makes the framework suitable for resource-constrained industrial and operational settings.

## Explainable AI analysis

**Fig. 15:**
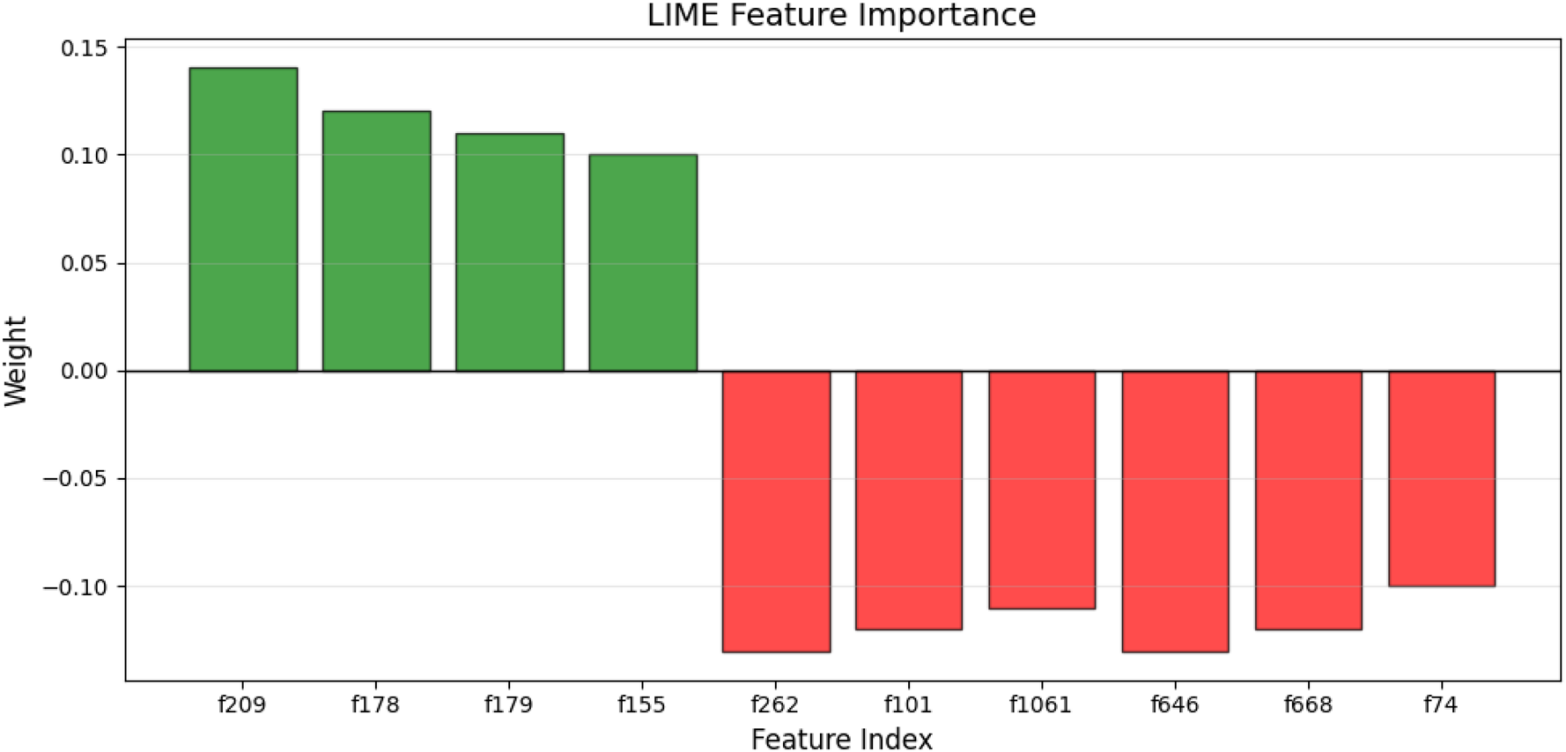
LIME-based local explanation for test sample 342.

A collection of methodologies termed explainable artificial intelligence (XAI) transforms the obscure internal logic of complex models—such as ensemble meta-classifiers, deep neural networks, and large language models—into comprehensible descriptions for humans, including feature attributions, saliency maps, or surrogate rules. XAI operates post-hoc, examining the impact of minor alterations in the input on the output, and subsequently synthesizing that behaviour into coherent, valuable explanations, rather than modifying the training weights. Engineers must ascertain the signal parameters utilized by the classifier, regulators need transparent audits, and users should have the ability to contest erroneous results, as misclassification in wireless communication networks may lead to spectrum interference or key operational failures. Consequently, explainability facilitates data-centric enhancement, focused model troubleshooting, adherence to regulations, and the establishment of confidence. To illustrate our methodology, we generated Local Interpretable Model-agnostic Explanations (LIME) for every test waveform and analysed the representative case shown in Figure 15. The fused representation combines three complementary feature families. MobileNetV2 contributes lightweight local texture and edge descriptors, ResNet-50 contributes deeper hierarchical semantic patterns, and InceptionV3 contributes multi-scale modulation-sensitive structures. The LIME analysis further indicates that the classifier primarily relies on stable amplitude-phase regions and constellation-density patterns, which are directly relevant to discriminating between phase-shift, amplitude-shift, and quadrature-amplitude modulation families. From a practical perspective, these explanations are useful in at least three ways. First, they help verify whether the model attends to modulation-relevant amplitude-phase structures instead of spurious artifacts. Second, they support debugging by identifying cases in which incorrect predictions are associated with unstable or diffuse highlighted regions. Third, they provide engineers with a transparent basis for trust in real-time deployment, especially when AMC predictions feed downstream decisions such as adaptive transmission, signal monitoring, or interference management.

We apply LIME in the image domain by segmenting the polar image into superpixels and learning a sparse local surrogate model around each test sample. The explanation highlights superpixels that most increase the probability of the predicted modulation. Figure 15 shows that the model relies on stable constellation-density regions and phase-amplitude structures rather than isolated pixels. To move beyond a single example, we aggregate explanations over 200 random test samples per class and report the most frequently selected superpixel regions, which reveals consistent decision patterns for each modulation family.

**Table 16:** Summary of aggregated LIME explanations: top superpixel regions frequently selected per modulation family (200 samples per class).

Modulation	Most frequent highlighted regions (description)
BPSK	Two antipodal clusters at 45$^\circ$ and 225$^\circ$ (or 0$^\circ$/180$^\circ$) on a single radius, highlighting binary phase separation.
QPSK	Four distinct quadrant clusters at 0$^\circ$, 90$^\circ$, 180$^\circ$, and 270$^\circ$, emphasizing quadrant phase stability.
8PSK	A uniform ring divided into 8 sector clusters, focusing on granular phase differences across signal constellation.
QAM family	Concentric rings (typically 2–3 levels) with multiple phase offsets per ring, distinguishing amplitude levels and quadrature points.
AM/FM family	Full circular density (FM) or radial lines (AM) indicating continuous signal variation rather than discrete clusters.

The near-balanced opposition between supportive and conflicting contributions indicates that the model synthesises evidence from diverse spectral indices rather than relying on a single dominant cue.

**Fig. 16:**
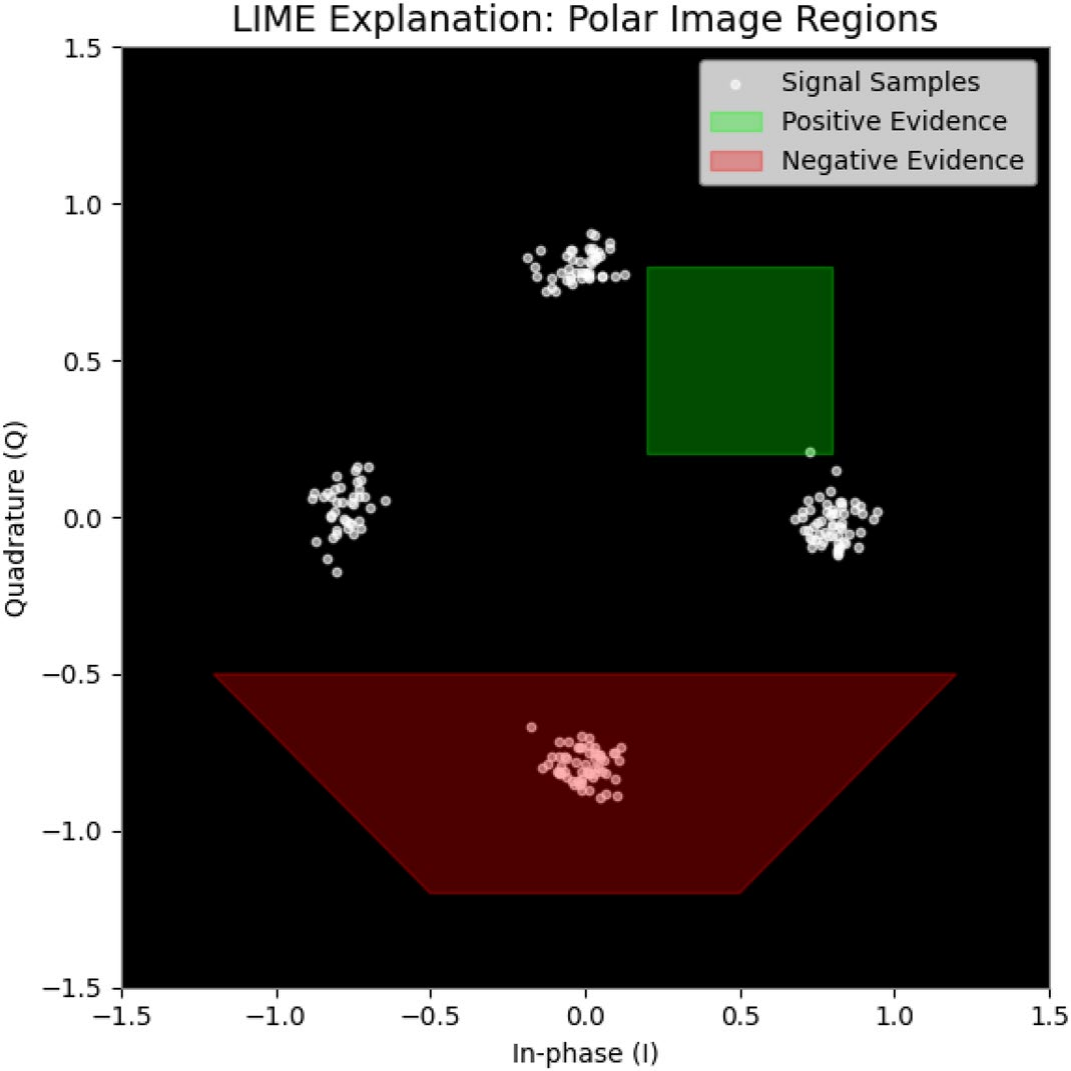
LIME explanmation: Polar Image Region.

This behaviour corresponds with the complementing characteristics of the Inception V3, ResNet-50, and MobileNetV2 embeddings. Thus, the model provides a clear, sample-specific explanation that facilitates the root-cause study of misclassifications and lays the groundwork for future worldwide audits via the utilization of SHAP or aggregated LIME scores.

## Conclusion

This paper proposed a robust and efficient framework for automatic modulation classification by leveraging deep feature representations from pretrained deep learning models like InceptionV3, ResNet50, and MobileNetV2 and feeding their concatenated features into an Extreme Learning Machine (ELM) optimized via Grey Wolf Optimization (GWO). The GWO-ELM mitigates local minima and facilitates accelerated convergence while exhibiting more simplicity compared to alternative classifiers. To improve transparency, we incorporate explainable AI methodologies named as LIME to elucidate and verify each classification conclusion. The model has been assessed in both standalone and cloud environments with diverse virtual machine configurations. The maximum classification accuracy of 95.16% was attained using the vCPU-16 64 GB RAM configuration. Future endeavours will involve the utilization of increased computational resources and the optimization of supplementary GWO-ELM parameters to further augment performance.

## Data Availability

The datasets used and/or analyzed during the current study available from the corresponding author on reasonable request.

## Abbreviations

AMC: Automatic Modulation Classification
CNN: Convolutional Neural Network
ELM: Extreme Learning Machine
GWO: Grey Wolf Optimization
XAI: Explainable Artificial Intelligence
LIME: Local Interpretable Model-agnostic Explanations
SNR: Signal-to-Noise Ratio
GAP: Global Average Pooling
FC: Fully Connected
*I*[*n*]: In-phase component
*Q*[*n*]: Quadrature component
*r*[*n*]: Polar amplitude
$$\theta [n]$$: Polar phase
$\textbf{P}$: Polar image matrix
$\textbf{z}$: Fused feature vector
*H*: Number of hidden neurons
$$\lambda$$: Ridge coefficient
